# Etiology of community acquired pneumonia among children in India: prospective, cohort study

**DOI:** 10.7189/jogh.05.020418

**Published:** 2015-12

**Authors:** Joseph L. Mathew, Sunit Singhi, Pallab Ray, Eva Hagel, Shanie Saghafian–Hedengren, Arun Bansal, Sofia Ygberg, Kushaljit Singh Sodhi, B V Ravi Kumar, Anna Nilsson

**Affiliations:** 1 Department of Pediatrics, PGIMER, Chandigarh, India; 2 Department of Medical Microbiology, PGIMER, Chandigarh, India; 3 Department of Learning, Informatics, Management and Ethics, Karolinska Institutet, Stockholm, Sweden; 4 Dept of Women’s and Children’s Health, Karolinska Institutet, Stockholm, Sweden; 5 Department of Radiodiagnosis and Imaging, PGIMER Chandigarh, India; 6 Xcyton Diagnostics Pvt Ltd, Bangalore, India

## Abstract

**Background:**

Childhood community acquired pneumonia (CAP) is a significant problem in developing countries, and confirmation of microbial etiology is important for individual, as well as public health. However, there is paucity of data from a large cohort, examining multiple biological specimens for diverse pathogens (bacteria and viruses). The Community Acquired Pneumonia Etiology Study (CAPES) was designed to address this knowledge gap.

**Methods:**

We enrolled children with CAP (based on WHO IMCI criteria of tachypnea with cough or breathing difficulty) over 24 consecutive months, and recorded presenting symptoms, risk factors, clinical signs, and chest radiography. We performed blood and nasopharyngeal aspirate (NPA) bacterial cultures, and serology (*Mycoplasma pneumoniae*, *Chlamydophila pneumoniae*). We also performed multiplex PCR for 25 bacterial/viral species in a subgroup representing 20% of the cohort. Children requiring endotracheal intubation underwent culture and PCR of bronchoalveolar lavage (BAL) specimens.

**Findings:**

We enrolled 2345 children. NPA and blood cultures yielded bacteria in only 322 (13.7%) and 49 (2.1%) children respectively. In NPA, *Streptococcus pneumoniae* (79.1%) predominated, followed by *Haemophilus influenzae* (9.6%) and *Staphylococcus aureus* (6.8%). In blood, *S. aureus* (30.6%) dominated, followed by *S. pneumoniae* (20.4%) and *Klebsiella pneumoniae* (12.2%). *M. pneumoniae* and *C. pneumoniae* serology were positive in 4.3% and 1.1% respectively. Multiplex PCR in 428 NPA specimens identified organisms in 422 (98.6%); of these 352 (82.2%) had multiple organisms and only 70 (16.4%) had a single organism viz. *S. pneumoniae*: 35 (50%), Cytomegalovirus (CMV): 13 (18.6%), Respiratory Syncytial Virus (RSV): 9 (12.9%), other viruses: 6 (8.7%), *S. aureus:* 5 (7.1%), and *H. influenzae*: 2 (2.9%). BAL PCR (n = 30) identified single pathogens in 10 (*S. pneumoniae*–3, CMV–3, *S. aureus*–2, *H. influenzae*–2) and multiple pathogens in 18 children. There were 108 (4.6%) deaths. The pattern of pathogens identified did not correlate with pneumonia severity or mortality.

**Conclusions:**

The majority of children with CAP have multiple pathogens (bacteria and viruses). *S. pneumoniae* and *S. aureus* predominate in NPA and blood respectively. CMV and RSV were the dominant respiratory viruses in NPA and BAL. The presence of multiple pathogens, especially organisms associated with nasopharyngeal carriage, precludes confirmation of a causal relationship in most cases.

Pneumonia is a leading cause of childhood morbidity and mortality globally. It is estimated that there were over 120 million episodes of pneumonia among children younger than five years during 2010–11; of which over 10% were severe episodes [1]. A recent systematic review estimated 0.22 pneumonia episodes per child–year in developing countries alone [2], with nearly one in eight cases progressing to severe disease. Yet another systematic review estimated nearly 12 million hospitalizations in 2010 due to severe pneumonia and 3 million due to very severe disease [3]. Pneumonia is also estimated to be responsible for almost 1 million deaths among children under 5 years old [4], with maximum burden in Africa and South Asia [3]. India has a high burden of childhood pneumonia and the disease accounts for about a quarter of the under–five mortality in the country [5]. Recognizing this burden, the World Health Organization (WHO) developed and disseminated a simple case definition for identification and treatment of pneumonia, which could be used by field–workers in resource–poor settings [6-9]. It relies on the physiological principle that parenchymal lung disease results in compensatory tachypnea; therefore any tachypnea indirectly indicates parenchymal disease including pneumonia. This case definition is highly sensitive, and does not require chest radiography.

Traditional teaching attributes most cases of childhood community acquired pneumonia (CAP) to a few micro–organisms, mostly bacteria [8]. In recent decades, developed countries have witnessed a shift from bacterial to viral predominance on account of hygiene, sanitation, infection control, and vaccination policies. Recent systematic reviews of childhood pneumonia etiology suggest that in developing countries, a few bacteria (*S. pneumoniae* and *H. influenzae*) and viruses (Respiratory Syncytial Virus, Influenza virus) are associated with majority of childhood CAP [3,5,10-12]. A systematic review from India suggested that about 15–24% of bacterial pneumonia in South Asian countries can be attributed to *S. pneumoniae* [13]. Similarly data from the Invasive Bacterial Infection Surveillance (IBIS) network in India suggests that invasive Pneumococcal disease could be a significant public health problem in the country, contributing to significant morbidity and mortality [14]. However these data were not based on studies designed to determine pneumonia etiology.

The Pneumonia Research for Child Health (PERCH) project [15] is a 7–site case–control study to identify the cause of pneumonia among children in developing countries. However, none of the sites is located in India. Pilot data from PERCH reported 152 potentially pathogenic isolates among 108 hospitalized cases, using multiple microbiologic techniques on various body fluids. Viruses represented over 80% of the pathogens detected [16].

Conventional methods for determining etiology, such as bacterial culture of blood or nasopharyngeal swabs, and/or selective application of serological tests for a few organisms, are limited by poor sensitivity, or low specificity, or both. On the other hand, diagnostic techniques with greater specificity are limited by technical difficulty, invasive procedures, and high cost.

Accurate, reliable and rapid determination of etiology in childhood CAP is important because it would influence individual treatment decisions, antibiotic policy in the community, and also rational immunization policy at a national level. Currently, there is no study from India reporting etiology of CAP in a large cohort of children, using multiple biological samples, and various sensitive as well as specific microbiologic methods. We initiated the Community Acquired Pneumonia Etiology Study (CAPES) to address this knowledge gap by determining the microbiologic etiology of CAP in a cohort of Indian children using multiple biological specimens (blood, nasopharyngeal aspirates, bronchoalveolar lavage) and the relationship between etiology and pneumonia severity.

## METHODS

This prospective study was carried out in the Union Territory of Chandigarh (located in north India with a population of 1.05 million residing in urban, rural and urban–slum areas, of whom 11.3% are children), over 24 consecutive months from 1 April 2011 to 31 March 2013. The study was coordinated from the Advanced Pediatrics Centre (APC) at PGIMER Chandigarh, a tertiary care centre with nearly 20 000 annual in–patient admissions and 100 000 out–patient visits.

**Figure 1:**
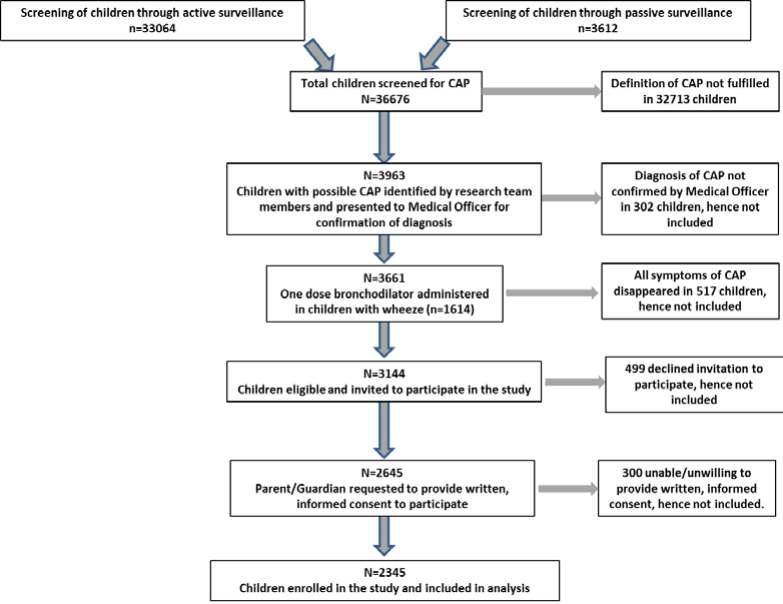
The screening process for children enrolled through passive or active surveillance. Trained research team members identified children with cough and /or difficult breathing, combined with tachypnea. If the child fulfilled WHO IMCI definition of CAP; confirmation of the diagnosis by a medical officer was required. Children whose symptoms of CAP disappeared with a single dose of bronchodilator were excluded. After obtaining written parental consent to participate, a total of 2345 children were enrolled in the study and included in analysis.

Enrolment of children aged 1 month to 12 years, fulfilling the WHO IMCI case definition of CAP designed for children <5 years [6-8], was carried out through active and passive surveillance (**Figure 1**). Tachypnea was defined as respiratory rate >60/min for infants <2 months; >50/min for infants 2–12 months; >40/min for children >12–60 months; and >30/min for children >60–144 months. Active surveillance was conducted in 30 anganwadi clusters, selected to represent the population of Chandigarh, where trained research team members visited households daily, inquiring for clinical symptoms of pneumonia. Passive surveillance was carried out by research staff stationed in the Out Patient and Emergency Departments of the APC, by evaluating clinical signs of CAP in children presenting to these Departments. If symptoms were reported and tachypnea confirmed, the child was presented to a Medical Officer for confirmation and inclusion. Children with duration of illness >7 days; those who had received antibiotics for >24 hours at presentation or those with previous hospitalization within the preceding 30 days, were excluded. Children with wheeze received a single dose of bronchodilator (Salbutamol 0.15mg/kg by nebulization), and those whose symptoms disappeared were excluded. All children received standard treatment including antibiotics, other medications as required and supportive care as per institution guidelines.

### Clinical work–up

Each child underwent a detailed history for demographic data, presence of risk factors for pneumonia, and immunization status. After physical examination, pneumonia severity was categorized based on the WHO classification [6-8]. In addition, all children underwent chest radiography. The radiographs were subsequently independently read by two trained investigators and scored as per the WHO criteria [17]. Discordant results were resolved through mutual discussion. In addition, children who required endotracheal intubation were also offered fiber–optic bronchoscopy and bronchoalveolar lavage (BAL), based on clinical need.

### Sampling and microbiological testing

A blood sample was drawn by venepuncture for routine investigations (hemogram, blood biochemistry). One to three ml blood was processed for bacterial culture using BACTEC 9240 (Becton Dickinson, Haryana, India) in Peds plus/F culture media (Becton Dickinson) [18]. The bottles were incubated at 37 °C for seven days and isolates were identified to species level by conventional biochemical and serological tests.

A nasopharyngeal aspirate (NPA) specimen was obtained from all children using a sterile, disposable suction catheter [19]. One aliquot was processed for bacterial culture and one aliquot was mixed with 3 ml saline and frozen at –80 °C for subsequent PCR analysis. BAL samples were similarly processed for bacterial culture and PCR. The Department of Medical Microbiology at PGIMER is accredited by the Government of India’s National Accreditation Board for Testing and Calibration Laboratories (NABL).

Serum was stored at –80 °C for *M. pneumoniae* and *C. pneumoniae* IgM serology performed using commercially available kits (Calbiotech Inc USA) according to the manufacturer’s instructions [20,21] and analyzed with an automated ELISA reader (SPECTROstar Nano, BMG LabTech, Germany) [22]. Serological tests were run in duplicate and only concordant results were labeled as positive or negative.

Multiplex PCR was performed on a subset of samples representing 20% of the cohort, selected through a randomization procedure stratifying by age, pneumonia severity and season. PCR was performed for detecting a panel of respiratory bacteria and viruses (Table S1 in **Online Supplementary Document(Online Supplementary Document)**) at Xcyton Diagnostics Pvt Ltd, Bangalore, also NABL accredited, using the Syndrome Evaluation System (SES) for Pneumonia. The SES was standardized to attain 100% sensitivity and specificity using quantified virus panels available from Quality Control for Molecular Diagnostics (QCMD), UK [23]. (Table S2 in **Online Supplementary Document(Online Supplementary Document)**). Limit of Detection for all DNA viruses was 250 virions/mL and 100 virions/mL for CMV and adenoviruses. For RNA viruses, QCMD proficiency panels of 2011 were used. Samples were thawed, centrifuged (3000 rpm×10 min) and re–suspended in 1 mL sample supernatant. Nucleic acids were extracted using commercially available Qiagen kits and cDNA was prepared using a commercial cDNA Archive Kit (ABI, USA) [24], both according to the manufacturer’s instruction with the addition of pathogen specific primers. Amplification was carried out in Bio–Rad PTC200 thermal cycler and the detection of amplified products was facilitated using biotin labeled primers. Samples were categorized as negative or positive for any pathogen with internal controls (human housekeeping genes β2–microglobulin and β–actin) included in each run as control for DNA and RNA extraction respectively.

### Statistical analysis

Descriptive statistics of cohort characteristics and duration of various symptoms are presented with proportional distribution and median (IQR) respectively. Ordinal categorical data and mortality status was analyzed using test of linear association. Data analysis was conducted in IBM SPSS Statistics 22.0 [25].

*Role of the funding source***:** The funding agency had no role in study design, data collection, data analysis, data interpretation, writing of the manuscript or decision to submit for publication. All authors had access to the data in the study and approved the decision to submit for publication.

## RESULTS

A total of 36 676 children underwent active or passive surveillance for CAP. **Figure 1** shows the step–wise process used to enrol children. A total of 2345 children were enrolled and comprised the cohort included in the analysis.

**Table 1** presents the baseline characteristics of children enrolled through active or passive surveillance. Children <12 months dominated in both groups. Severe and very severe disease was detected more frequently in children enrolled through passive surveillance. A total of 1145/2345 children (48.8%) were enrolled during the cold season from 16 November to 15 February; while the remaining (51.2%) were enrolled during the longer warm season. Acute malnutrition, defined as weight–for–age z score less than 3, was observed in 1008/2345 children (42.9%). Similarly, absent or deficient breastfeeding (defined as duration of breastfeeding <6 months for infants older than six months, or less than infant’s age in those <6 months old) was more common in those enrolled through passive surveillance. These children were also more likely to be exposed to solid fuels as well as tobacco smoke in their homes. There were no major differences in gender, history of wheeze, previous history of infections, or family history of tuberculosis in children enrolled through active or passive surveillance.

**Table 1:** Baseline characteristics of children enrolled in the study*

		Active surveillance			Passive surveillance	
		n = 746	%		n = 1599	%
**Gender:**						
Male		558	74.8		1123	70.2
**Age group:**						
1–2 months		10	1.3		142	8.9
3–12 months		295	39.5		887	55.5
13–60 months		382	51.2		424	26.5
61–144 months		59	7.9		146	9.1
**Severity:**						
Pneumonia		609		81.6	424	26.5
Severe pneumonia		131		17.6	870	54.4
Very severe pneumonia		6		0.8	305	19.1
**Season:**						
Cold		360		48.3	785	49.1
Warm		386		51.7	814	50.9
**Malnutrition**		249		33.4	759	47.5
**Absent or deficient breast feeding**		63		8.4	230	14.4
**History of wheezing**		112		15.0	200	12.5
**History of >1 episodes of URI and/or diarrhea**		207		27.7	415	26.0
**Current or previous family history of TB**		28		3.8	49	3.1
**Past history of TB**		11		1.5	25	1.6
**Predominant use of solid fuel**		223		29.9	764	47.8
**Any use of solid fuel**		257		34.5	816	51.0
**Exposure to tobacco smoke at home**		172		23.1	489	30.6

URI – Upper respiratory infections, TB – tuberculosis

*See Table S3 in **Online Supplementary Document(Online Supplementary Document)** for definitions.

**Table 2** presents symptoms reported by parents, clinical findings and radiography. Almost all children presented with cough, fever and fast breathing with median duration of symptoms being similar in those enrolled through active or passive surveillance. Parents reported wheezing during the current episode in approximately one-third of the children. Symptoms/signs suggesting greater severity of pneumonia were more frequently identified in those enrolled through passive surveillance. A larger proportion of these children also had WHO Class I and Class II chest X–rays.

**Table 2:** Presenting symptoms, clinical examination findings and chest radiography at enrolment into the study

	Active surveillance		Passive surveillance	
	n = 746	%	n = 1599	%
Symptoms at presentation:				
Fast breathing	698	93.6	1556	97.3
– median duration in days (IQR):	2 (1–3)		2 (1–3)	
Cough	738	98.9	1459	91.2
– median duration (IQR):	4 (3–7)		4 (2–7)	
Fever	545	73.1	1254	78.4
– median duration (IQR):	3 (2–5)		3 (2–5)	
Difficult breathing	412	55.2	1351	84.5
– median duration (IQR):	2 (1–3)		2 (1–4)	
Chest indrawing	156	20.9	1097	68.6
– median duration (IQR):	2 (1–3)		2 (1–3)	
Wheezing	239	32.0	621	38.8
– median duration (IQR):	2 (2–3)		2 (1–3)	
Altered mental status	60	8.0	395	24.7
Inability to drink	29	3.9	350	21.9
**Clinical findings:**				
Pallor	48	6.4	398	24.9
Cyanosis	6	0.8	101	6.3
Retractions	193	25.9	1178	73.7
Crackles	476	63.8	1225	76.6
Wheezing	289	38.7	553	34.6
**Oxygen saturation:**				

**>95%**	**658**	**88.2**	**802**	**50.2**
92–95%	65	8.7	402	25.1
<92%	23	3.1	393	24.6

Radiography findings:*				
WHO Class I	272	36.8	770	48.5
WHO Class II	179	24.2	482	30.4
WHO Class III	285	38.6	323	20.4
WHO Class IV	3	0.4	12	0.8
**Mortality**	1	0.1	107	9.2

IQR – interquartile range

*WHO categorization of chest radiography [17]: Class I = consolidation/pleural effusion; Class II = interstitial pattern/infiltrate; Class III = no consolidation/ infiltrate/ effusion; Class IV = radiograph quality not sufficient for reading.

There were 108 (4.6%) deaths; of these 107 occurred among those enrolled through passive surveillance (mortality rate 9.2%) and one among those enrolled through active surveillance (0.1%). Based on disease severity, the mortality rate was 1.2% for pneumonia, 4.7% for severe pneumonia and 15.8% for very severe pneumonia. A comparison between fatal and non–fatal cases suggested that age <12 months, oxygen saturation <95% and radiographic finding of consolidation (WHO Class I) were associated with mortality.

**Table 3** summarizes the microbiology findings from culture of different biological specimens. Blood culture results were available in 2285 children (97.4%) and only 49 (2.1%) were positive for pathogenic species. NPA culture results were available in 2323 children (99.1%) and of these, potentially pathogenic organisms were identified in 322 (13.7%). BAL culture (performed in children requiring endotracheal intubation), 0–5 days (median 2 days) after enrolment was positive in only 3/30 children. In addition, serology was positive for *M. pneumoniae* in 103 (4.3%) and *C. pneumoniae* in 26 (1.1%).

**Table 3:** Bacterial culture in clinical specimens

Organism	Blood (n = 2285)	NPA (n = 2323)	BAL (n = 30)
*S. aureus*	15	22	1
*S. pneumoniae*	10	255	1
*H. influenzae*	4	31	–
*K. pneumoniae*	6	3	–
Acinetobacter spp*	5	1	1
*S. typhi*	3	–	–
*Enterobacter spp*	1	–	–
*E coli*	1	3	–
*Pseudomonas spp*	–	4	–
*Stenotrophomonas maltophila*	–	1	–
Yeast spp	–	1	–
Multiple	4†	1‡	–
**Total**	**49**	**322**	**3**

NPA – nasopharyngeal aspirate, BAL – broncho-alveolar lavage

*Acinetobacter Baumanii and *Lwofii*.

†Two children had *S. pneumoniae* + *S. aureus*; and one child each had *S. aureus* + *Enterococcus faecalis* and *Pseudomonas* + *E coli*

‡One child had *Acinetobacter spp* + *K. pneumoniae*

**Figure 2:**
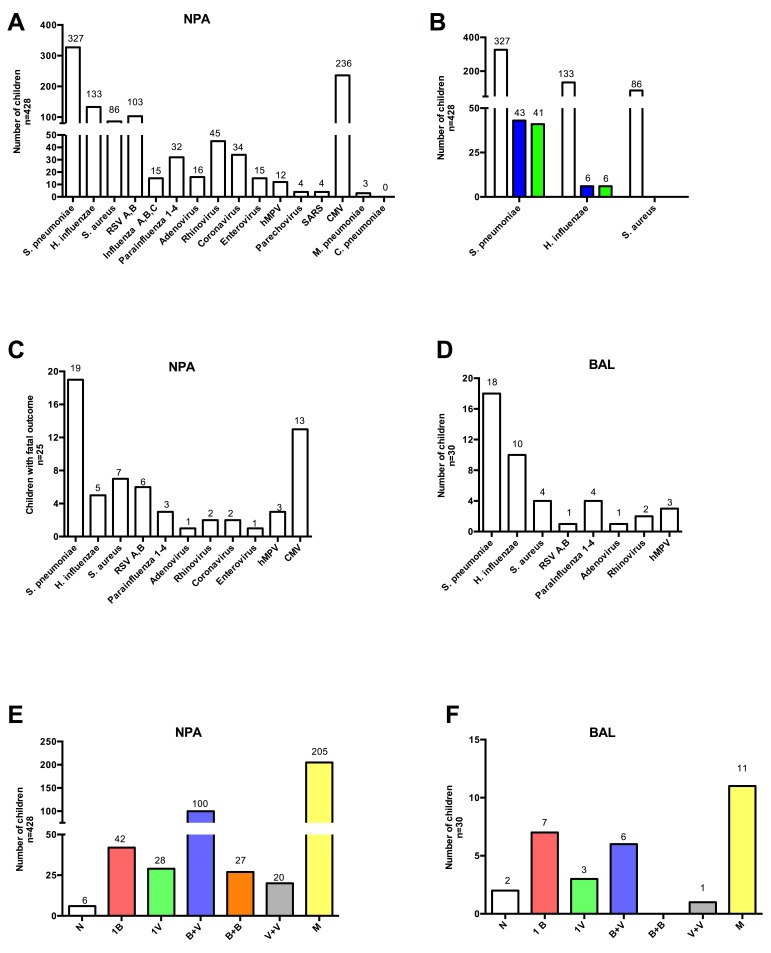
Microbiological findings in samples obtained from the sub–group (n = 428) of children with CAP. The number above each bar represents the number of children with a positive result. **(A)** Nasopharyngeal aspirate (NPA) Multiplex–PCR findings (bacteria and viruses) in the sub–group (n = 428). (**B**) Comparison of diagnostic yield of bacteria in NPA by PCR and culture indicates that PCR has a higher sensitivity; PCR (white bars), NPA (blue bars) and double positive samples (green bars). **(C)** NPA PCR findings in **c**hildren with fatal outcome (n = 25). (**D**) BAL PCR findings in children who were intubated and underwent broncho–alveolar lavage (n = 30). Combinations of pathogens in **(E)** NPA samples (n = 428) and **(F)** BAL samples (n = 30). N – Nil, B – Bacteria, V – Virus, M – multiple organisms.

**Figure 2** summarizes the data from microbiologic analysis of respiratory specimens. Of the 469 NPA samples selected for multiplex PCR, only 428 (91%) could be fully processed and 422 samples (98.6%) yielded organisms (panel A in **Figure 2**). A single bacterium or a single virus was found in only 42 (9.8%) and 28 (6.5%) children respectively. *S. pneumoniae* dominated (n = 35) followed by *S. aureus* (n = 5) and *H. influenzae* (n = 2). The single viruses identified were CMV (n = 13) and RSV (n = 9) followed by Rhinovirus (n = 2), and one each of Influenza, Parainfluenza, Enterovirus and hMPV. *S. pneumoniae* was the dominant organism identified in NPA culture as well. A comparison of the bacterial yield from NPA by PCR and culture is shown in panel B in **Figure 2**.

Among the 428 children with NPA PCR results, 25 died and PCR showed diverse organisms distributed in a pattern similar to the 428 children (panel C in **Figure 2**). Among intubated children undergoing bronchoscopy as part of clinical care (n = 30), only 2 samples were negative on PCR and the remainder showed organisms in a similar pattern to NPA PCR (panel D in **Figure 2**).

Since most NPA PCR samples yielded multiple pathogens, the data were analyzed with respect to etiology patterns rather than individual pathogens. These included combinations of two bacteria, two viruses, one bacterium plus one virus, or mixed i.e more than one bacteria and/or virus (panel E in **Figure 2**). The most common combination of pathogens in individual samples was *S. pneumoniae* and CMV (n = 100) followed by 2 bacteria or 2 viruses.

In BAL samples, the single pathogens identified were *S. pneumoniae* (n = 3), *S. aureus* (n = 2), *H. influenzae* (n = 2) and CMV (n = 3); the majority of samples (n = 18) showed multiple organisms (panel F in **Figure 2**) that were distributed in a pattern almost similar to NPA samples.

**Figure 3:**
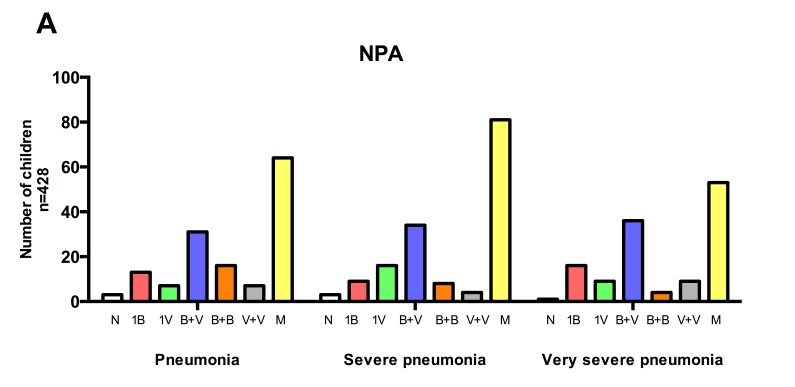
NPA Multiplex–PCR findings stratified by pneumonia severity as defined by WHO IMCI criteria in the sub cohort (n = 428). N – Nil, B – Bacteria, V – Virus, M – multiple organisms.

The complex microbial patterns on PCR were further analyzed with respect to disease severity (defined according to WHO criteria) but there were no apparent differences (**Figure 3**).

## DISCUSSION

To our knowledge, this is one of the largest single–centre studies of CAP etiology in children from a resource–limited setting. Our data suggest that CAP is associated with a number of pathogens or combinations of viral and bacterial pathogens. Further, no single pathogen or combination could be related to disease severity. Our findings also confirm that infants <12 months old are particularly vulnerable in terms of disease severity and outcome.

As expected, *S. pneumoniae* was the predominant isolate in NPA by culture as well as PCR, although mere detection does not establish a causal role. We could not do serotyping due to resource constraints. It can be argued that the isolation rate by culture in our cohort is lower than expected [26,27], especially as PCR identified *S. pneumoniae* much more frequently. It is possible that clinical pneumonia due to other pathogens masks the presence of *S. pneumoniae* on routine culture. The major difficulty in attributing etiology to *S. pneumoniae* is its frequent presence in asymptomatic children also, although a similar argument could be raised for *S. aureus* too [28,29].

Detection of multiple pathogens in NPA by PCR makes it difficult to ascribe a causal role to any one organism. Our culture and PCR data also suggest that nasopharyngeal specimens may perhaps be inappropriate for confirming microbial etiology in CAP. Indeed, this is in concordance with several recent studies showing the presence of various viruses in asymptomatic children as well as those with upper respiratory tract symptoms [19,30,31]. It appears that even *M. pneumoniae* can be identified in the nasopharynx of healthy children [32].

Somewhat surprisingly, CMV was the most common virus in our cohort, where none had immune–suppressive therapy, known primary immune–deficiency and where the HIV prevalence during the study period is reported to be <0.25% in the community [33]. While CMV is well–recognized as a pathogen in these latter settings, its frequent occurrence in CAP raises the possibility that it may contribute to pneumonia pathogenesis singly or with other pathogens [34]. This novel finding also emphasizes that although PCR is highly sensitive, it can detect only those organisms that are looked for–a limitation that is being overcome by next generation sequencing. After CMV, RSV was most frequently identified as previously reported also [2] while Influenza A and B were less frequent. Unfortunately, even BAL samples in a limited number of children could not ascertain etiology as most children had multiple organisms. Further the time–lag between presentation and obtaining BAL samples in the majority of children raises the possibility that some of the organisms could represent secondary infection.

How to interpret the detection of multiple organisms in respiratory tract samples from a given child? It is possible that infection by one (potential) pathogen facilitates other pathogens, or that mild infection with one organism becomes more severe in the presence of additional organisms. This is well documented with Influenza infection [35,36] and suggested for other organisms also [2]. However, the pattern of PCR findings did not differ with disease severity which is in concordance with initial data from the PERCH project also [16]. In our cohort, a single organism (bacteria or virus) was identified by NPA PCR in only a minority of children. Further NPA data may be skewed on account of nasopharyngeal carriage. The limited BAL data suggests that *S. pneumoniae*, CMV, *S. aureus* and *H. influenzae* may be the dominant pathogens in severe cases of CAP. In children with fatal outcome, the same pathogens were identified along with RSV.

In the small number of positive blood cultures, *S. aureus* predominated, rather than *S. pneumoniae* or *H. influenzae*, expected in a vaccine–naïve pediatric population such as our cohort. Clinical experience suggests that *S. aureus* is frequently responsible for community acquired infections in India, although it has not previously been documented as the most frequent cause of bacteraemia in childhood pneumonia. In contrast, it is the most frequently recovered pathogen in parapneumonic effusions/empyema complicating pneumonia [37-39] and also commonly isolated in blood cultures from infants with bacteraemia [40]. Therefore it is reasonable to conclude that *S. aureus* may be an important pathogen in childhood pneumonia as well. However, international and national antibiotic treatment protocols for childhood CAP do not use specific antibiotics against this organism.

The data presented in this study raise some important points for further research on childhood CAP. First, the mere identification of organisms by highly sensitive techniques may not confirm etiology. Even comparing the yield among cases vs controls, as planned in the PERCH Project [41] can at best suggest an association, but not causation. In an individual child, even the presence of organisms commonly associated with pneumonia may be of limited value for predicting pneumonia severity/outcome. The presence of potential pathogens in the respiratory secretions of apparently healthy children also raises the possibility that microbes may not be solely responsible for disease. It is likely that combinations of host immune status and/or response to infection/inflammation tip the balance from asymptomatic colonization to disease in a given child.

Although this study had several methodological strengths limiting the risk of bias, it also had limitations. The disproportionately large number of severe and very severe pneumonia cases attest to greater enrolment through passive surveillance. Lack of controls is a limitation since it would have provided data on nasopharyngeal carriage of pathogens in asymptomatic/ healthy children in this population. Further, research team members could not be stationed in a given anganwadi throughout the study period, hence pneumonia incidence could not be calculated. We could perform only qualitative PCR, and that too in a small proportion (20%) of the cohort.

## CONCLUSION

This large cohort study (CAPES) identified multiple pathogens in various biological samples of children with CAP. Our data suggest that it is difficult to attribute etiology to a single pathogen in the majority of cases as co–infection is common and independent of disease severity. Multiplex PCR proved to be highly sensitive in identifying potential pathogens from respiratory samples; but lacked specificity for establishing a causal relationship. A novel finding of CMV carriage/infection in nasopharyngeal secretions was observed. Our findings suggest that clinical practice guidelines for management of suspected bacterial pneumonia in developing countries should additionally consider anti–Staphylococcal therapy. Rational vaccination policies against *S. pneumoniae, H. influenzae* and (in the future) RSV could decrease overall burden of childhood pneumonia morbidity and mortality.
