# Supplementary material for: Etiology of community acquired pneumonia among children in India: prospective, cohort study
Source: J Glob Health. 2015 Oct 21;5(2):050418. doi: 10.7189/jogh.05.020418 (PMC4623579; doi:10.7189/jogh.05.020418)
Supplement: Online Supplementary Document [file jogh-05-020418-s001.pdf]

Online Supplementary Document

Mathew et al. Etiology of community acquired pneumonia among children in India: prospective, cohort study

J Glob Health 2015;5:020418

Table S1. Pathogens identified through multiplex-PCR

**Bacteria**	**Viruses**
S. pneumoniae	Influenza A,B,C
H. influenzae	Parainfluenza 1,2,3,4
S. aureus	RSV A,B
	Rhinoviruses
Atypical organisms	Enteroviruses
Mycoplasma pneumoniae	Coronaviruses OC43, 229E, NL63, HKU1
Chlamydophila pneumoniae	Human metapneumovirus
	Parechovirus
	Severe Acute Respiratory Syndrome (SARS) virus
	Adenovirus
	Cytomegalovirus

Table S2. Standardisation of PCR methodology for viruses and atypical organisms

**Viruses**
Influenza A,B,C: QCMDINFRNA11
Parainfluenza 1,2,3,4
RSV A,B: QCMDRSVRNA11
Rhinoviruses: QCMD RVRNA11
Enteroviruses
Coronaviruses OC43, 229E, NL63, HKU1: QCMD CVRNA11
Human metapneumovirus: QCMD MPVRNA2011
Parechovirus: QCMD 2013 Parechovirus RNA EQA programme
Severe Acute Respiratory Syndrome (SARS) virus: QCMD CVRNA11
Adenovirus: QCMD 2013 Adenovirus DNA EQA Programme,
Cytomegalovirus: QCMD 2013 Cytomegalovirus DNA EQA programme,
Atypical organisms
Mycoplasma pneumoniae: QCMDCP-MP12
Chlamydophila pneumoniae: QCMDCP-MP12

Table S3. Definitions of terms used in this study

• Pneumonia was defined as per the IMCI definition [5-7] as cough or difficult breathing associated with fast breathing/tachypnea. Tachypnea was defined as Respiratory rate (counted twice for one full minute by trained research team members and re-confirmed by a trained medical officer) >60/min for infants <2 months; >50/min for infants 2-12 months; >40/min for children >12-60 months; and >30/min for children >60-144 months.
• Severe pneumonia was defined as the presence of lower chest indrawing in a child with pneumonia.
• Very severe pneumonia was defined by the presence of any general danger sign (as per the IMCI criteria) such as inability to feed, dull/unresponsive, vomiting everything, seizures, central cyanosis or unconsciousness.
• Cold season was defined from 16 November to 15 February; and Warm season from 16 February to 15 November.
• Acute malnutrition was defined as weight-for-age z score <-3
• Absent or deficient breastfeeding was defined as duration of breastfeeding <6 months for infants older than six months; or less than infant’s age for those <6 months old.
• Upper respiratory infection was defined as symptoms of common cold for less than 5 days duration viz runny nose, sneezing, dry cough, sore throat, with or without fever.
• Family history of TB was defined as diagnosed TB or history of taking anti-tuberculosis medication, in any family member/household contact.
• Predominant use of solid fuel was defined as usage of solid fuel (wood, coal or dried animal dung) as the main source of fuel used for cooking/heating purposes.
• Any use of solid fuel was defined as any usage of the above three solid fuels.
• Exposure to tobacco smoke at home was defined as current exposure to any family member/household contact smoking (any form of tobacco) indoors.
• WHO categorization of chest radiography [Q]**:** **Class I** = consolidation/pleural effusion; Class **II** = interstitial pattern/infiltrate; **Class III** = no consolidation/infiltrate/effusion; **Class IV** = radiograph quality not sufficient for reading.
